# Late gadolinium enhancement in mid infero-septal area after left bundle branch area pacing in the setting of bifascicular block and syncope: a case report

**DOI:** 10.1093/ehjcr/ytaf035

**Published:** 2025-01-23

**Authors:** Betim Redzepi, Samir Bengueddache, Juerg Schwitter, Panagiotis Antiochos, Etienne Pruvot

**Affiliations:** Department of Cardiology, Lausanne University Hospital (CHUV), Rue du Bugnon 46, 1005 Lausanne, Switzerland; Department of Cardiology, Lausanne University Hospital (CHUV), Rue du Bugnon 46, 1005 Lausanne, Switzerland; Department of Cardiology, Lausanne University Hospital (CHUV), Rue du Bugnon 46, 1005 Lausanne, Switzerland; Faculty of Biology and Medicine, University of Lausanne (UNIL), Quartier Centre, 1015 Lausanne, Switzerland; Department of Cardiology, Lausanne University Hospital (CHUV), Rue du Bugnon 46, 1005 Lausanne, Switzerland; Department of Cardiology, Lausanne University Hospital (CHUV), Rue du Bugnon 46, 1005 Lausanne, Switzerland; Faculty of Biology and Medicine, University of Lausanne (UNIL), Quartier Centre, 1015 Lausanne, Switzerland

**Keywords:** Syncope, Bifascicular block, Premature ventricular contractions, Orthostatic hypotension, Left bundle branch area pacing, Septal fibrosis, Case report

## Abstract

**Background:**

Conduction system pacing, which includes His bundle pacing and left bundle branch area pacing (LBBAP), is becoming increasingly common in clinical practice. His bundle pacing, introduced over two decades ago, continues to see growing use, while LBBAP is gaining traction due to its broader target area and shorter procedure times. However, complications such as septal perforation and lead-related issues may still arise. This case report explores the evaluation and management of recurrent syncope in the context of bifascicular block (BFB), highlighting the importance of thorough assessment, continuous ECG monitoring, and the potential need for permanent pacemaker implantation.

**Case summary:**

A 60-year-old woman with a history of myocardial infarction, Type II diabetes, and dyslipidaemia presented with recurrent syncope and BFB, for which a LBBAP device was implanted in August 2023. In February 2024, she was admitted again due to recurrent syncope and frequent premature ventricular contractions. Cardiac magnetic resonance imaging revealed two localized myocardial scars: one at the basal anterior wall from the 2018 ischaemic event and another in the mid infero-septal wall at the site of the LBBAP lead. During her hospital stay, she experienced multiple episodes of near fainting due to orthostatic hypotension, indicative of underlying autonomic neuropathy.

**Discussion:**

This narrative explores the evolving landscape of cardiac pacing techniques, with a focus on LBBAP, highlighting its advantages over traditional methods while also acknowledging potential complications. Additionally, it emphasizes the importance of a comprehensive workup for syncope and the need for tailored interventions that extend beyond pacemaker implantation.

**Learning points**

- The complexity of syncope presentation often requires a nuanced approach, and comprehensive history taking remains the cornerstone of determining the underlying cause.
- Syncope, even in the presence of bifascicular block, requires comprehensive evaluation to identify underlying causes and facilitate a tailored treatment approach.
- Left bundle branch area pacing restores left ventricular synchronization at the expense of limited complications, such as fibrosis that may lead to septal perforation, lead dislodgement, or loss of capture.

## Introduction

His bundle pacing (HBP) was first introduced over two decades ago, and its use has been increasing in recent years, supported by advancements in implantation tools.^1^ Left bundle branch area pacing (LBBAP), a more recent innovation, is gaining popularity rapidly due to its broader target area, shorter procedural times, and improved lead thresholds and electrical parameters.^2^ However, as with any medical intervention, complications such as intraprocedural or postoperative septal perforation,^3^ lead dislodgement,^4^ and loss of capture^5^ have been reported.

We present the case of a patient with syncope, frequent premature ventricular contractions (PVCs), and bifascicular block (BFB), who experienced recurrent syncope despite LBBAP implantation.

## Summary figure

**Figure:**
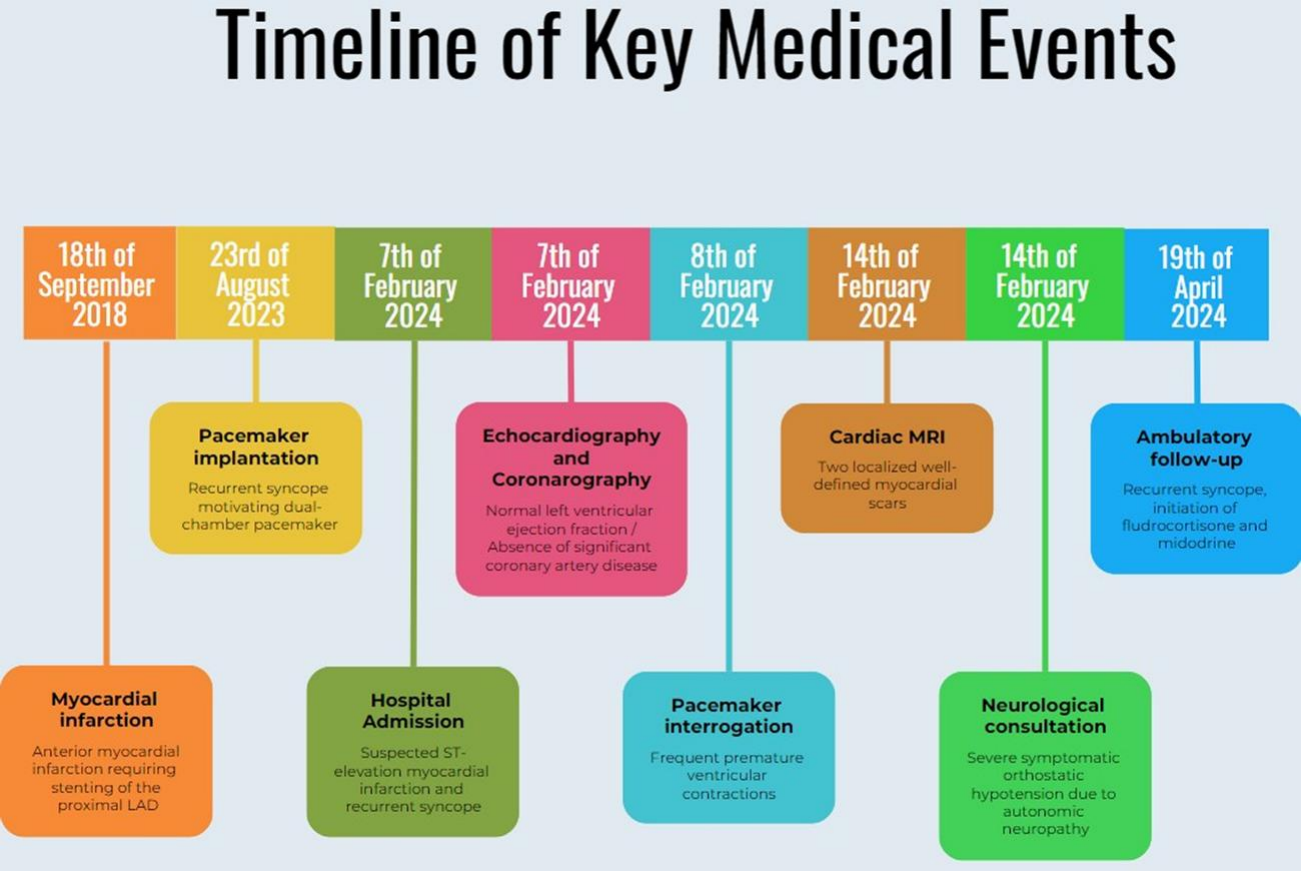


## Case summary

We present the case of a 60-year-old woman with a history of anterior myocardial infarction treated with stenting of the proximal left anterior descending artery in September 2018. Her comorbidities included poorly controlled Type II diabetes (HbA1C 9%) and dyslipidaemia. In August 2023, the patient began experiencing recurrent syncope. A cardiorespiratory physical examination revealed no abnormalities. Her ECG showed a BFB, leading to the implantation of a dual-chamber pacemaker, with leads placed in the right atrium and intraseptally at the left bundle branch. It should be noted that the patient did not undergo a cardiac magnetic resonance imaging (MRI) before the device implantation.

**Figure 1:**
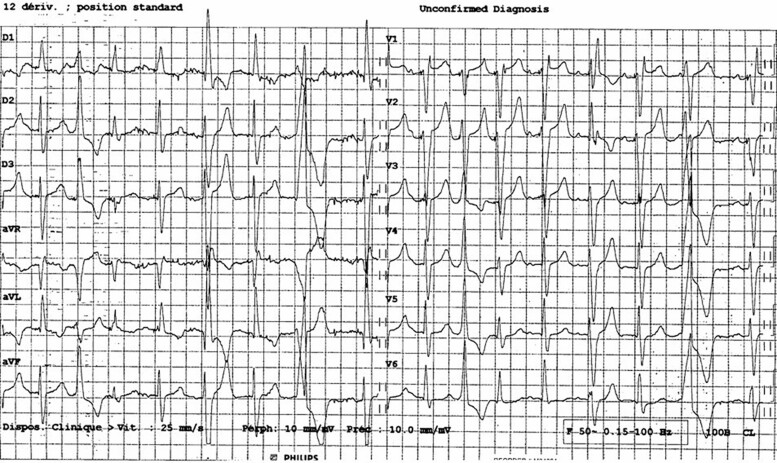
A 12-lead electrocardiogram demonstrating frequent ventricular premature contractions originating from the left ventricle outflow tract.

**Figure 2:**
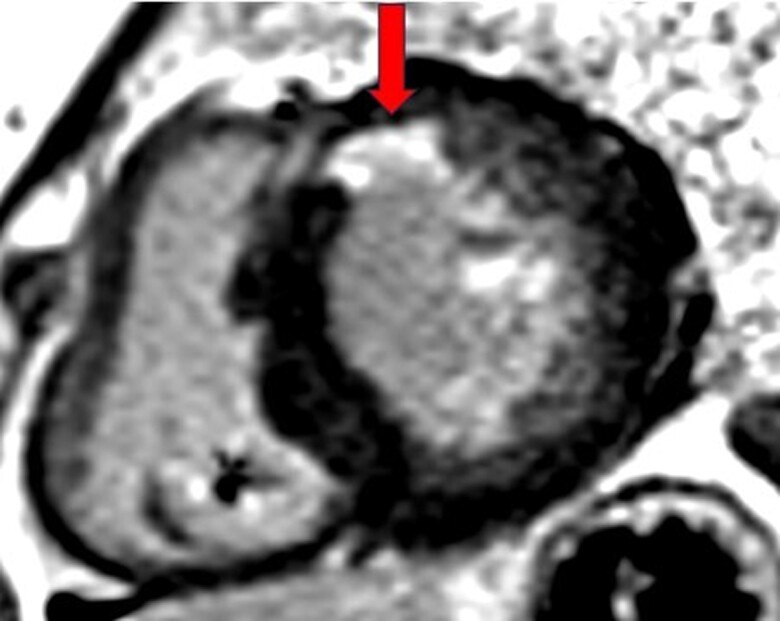
Post-contrast cardiac magnetic resonance imaging revealing subendocardial late gadolinium enhancement of the anterior basal myocardial segments in basal short-axis view (red arrow).

**Figure 3:**
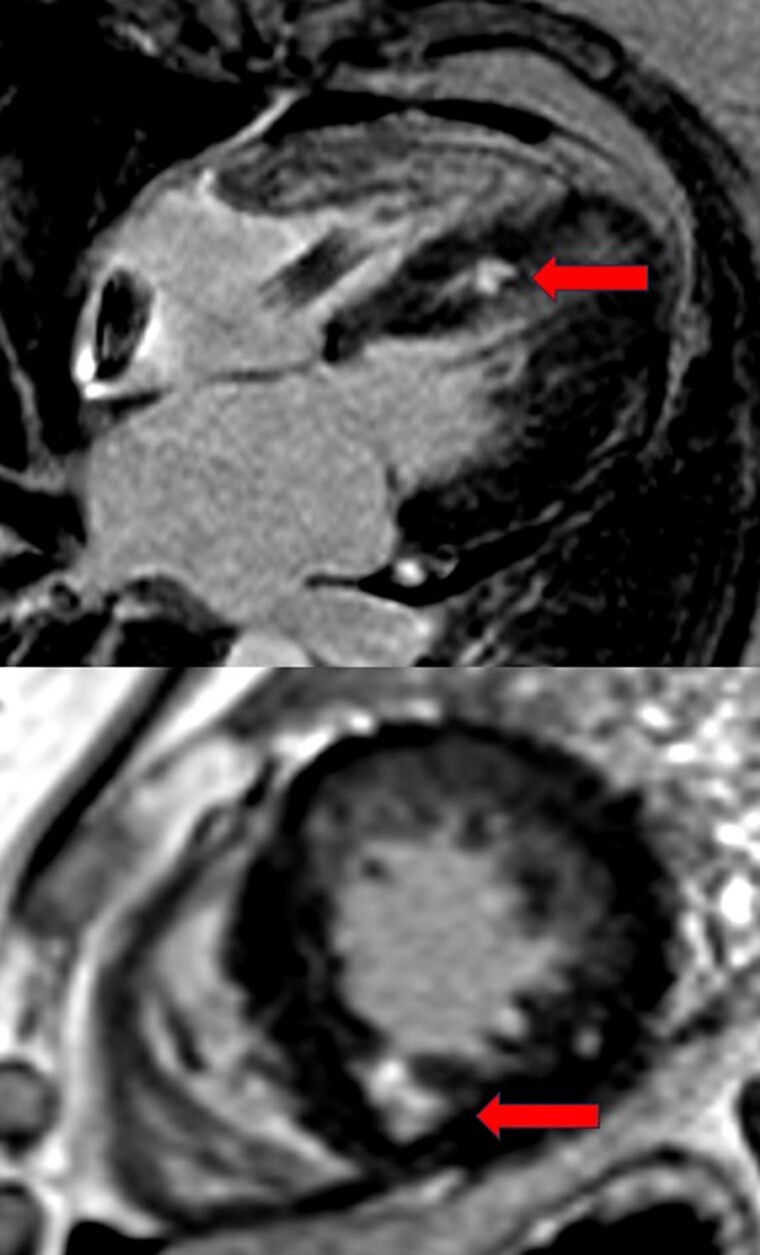
Post-contrast cardiac magnetic resonance imaging displaying subendocardial late gadolinium enhancement at the mid infero-septal myocardial segments in four-chamber long-axis view (top) and in mid short-axis view (bottom), precisely at the location of the left bundle branch area pacing lead (red arrows).

The patient was admitted to our hospital in February 2024 with suspected ST-elevation myocardial infarction and syncope following poorly defined chest discomfort and intermittent dyspnoea. The ECG showed an electrically paced ventricular rhythm with narrow QRS complexes. Upon admission, the patient no longer reported chest pain, but frequent PVCs originating from the antero-basal area were observed (*Figure 1*). The pacemaker interrogation showed stable parameters compared with those at implantation and did not reveal any sustained ventricular arrhythmias; however, it should be noted that the detection zone was set below the threshold for possible slow ventricular tachycardia. It is worth noting that prolonged Holter monitoring had previously been conducted from 30 to 31 August 2023, revealing 21% PVCs along with episodes of bigeminy and trigeminy. An echocardiogram revealed a normal left ventricular ejection fraction of 61% despite basal and mid antero-septal hypokinesis. Coronary angiography showed no significant stenosis, including in the proximal left anterior descending artery, where the previously placed stent remained patent. A cardiac MRI using a 1.5 T scanner (Siemens Healthineers Magnetom Sola) identified two localized, well-defined myocardial scars. The first scar was located in the subendocardium of the basal anterior wall, corresponding to the ischaemic event in 2018 (*Figure 2*). Interestingly, a second remote intramyocardial scar was found in the mid infero-septal wall of the left ventricle at the site of the LBBAP lead placement (*Figure 3*), which does not correspond to an ischaemic lesion based on the coronary angiography results and the intramural (not subendocardial) scar pattern observed on cardiac MRI.

During the hospital stay, the patient experienced multiple episodes of presyncope and an orthostatic test revealed a marked drop in blood pressure (from 123/53 to 77/24 mmHg), strongly suggestive of autonomic neuropathy. This finding contrasted with the test performed in the emergency room at the first hospital. This diagnosis was subsequently confirmed through a detailed neurological evaluation, including testing for peripheral neuropathy and reflex testing, which supported the presence of autonomic involvement. These findings, in combination with the patient’s clinical history and symptoms, led to the diagnosis of autonomic neuropathy. Further advanced testing, such as autonomic reflex screening or small fibre neuropathy tests, could be considered, but these were not deemed necessary in this case due to the clear clinical evidence of autonomic dysfunction.

## Discussion

Bifascicular block, a distinct pattern observed on the surface ECG, occurs when normal conduction through the His-Purkinje system is disrupted. This conduction disturbance typically manifests as a combination of right bundle branch block and either left anterior fascicular block or left posterior fascicular block, indicating that two of the three fascicles are impaired. It is also important to note that left bundle branch block is classified as a form of BFB.

In patients presenting with syncope or presyncope alongside advanced conduction disease, continuous inpatient ECG monitoring is typically performed for 24–48 h to detect high-grade atrioventricular (AV) block, which may necessitate permanent pacemaker implantation.^6^ For those with a structurally normal heart and unexplained syncope accompanied by BFB, an electrophysiologic study (EPS) can reveal occult infranodal conduction disease, potentially leading to the consideration of a permanent pacemaker. Patients with abnormal EPS findings, such as His-Ventricular interval > 70 ms or His-Purkinje AV block induced by pacing or pharmacologic challenge, often benefit from pacemaker implantation.^7^ For individuals with unexplained syncope and no clear cause, long-term monitoring with an insertable cardiac monitor is recommended.^6^ Cardiac pacing may also be considered for patients with unexplained syncope following a comprehensive evaluation and the presence of BFB.^6,7^ Currently, conduction system pacing (CSP) is primarily used for bradycardia indications. However, HBP can be associated with higher capture thresholds at implantation and, in some cases, a loss of conduction system capture during follow-up, which may lead to an increased rate of lead revisions compared with traditional pacing methods^8^ and syncope recurrence. Left bundle branch area pacing, using a ventricular intraseptal approach, has emerged as an alternative for achieving physiological pacing when HBP is not feasible. It is now frequently considered a first-line approach for delivering physiological pacing. Left bundle branch area pacing offers several practical advantages over HBP, including lower and more stable capture thresholds.^9,10^ Despite these benefits, LBBAP is not yet included in the European guidelines^7^ for pacing therapy, due to the limited data available at the time those guidelines were developed.

Jastrzębski *et al*.^11^ conducted the largest study to date on multicentre outcomes of LBBAP, involving 2533 patients across 14 European centres. They concluded that LBBAP is feasible as a primary pacing strategy for various indication, although implantation can be more challenging in patients with heart failure with reduced ejection fraction and prolonged QRS duration. While complications associated with the intraseptal lead route are not uncommon, most are minor, underscoring the need for further advancements in implantation tools and techniques. Following this, Burri *et al*.^12^ published a European Heart Rhythm Association clinical consensus statement on CSP implantation, which outlines standardized procedures aimed at improving technique and minimizing perioperative and postoperative complications. Among the complications mentioned are delayed septal perforation (0.1–0.3%), lead dislodgement (0.3–1.5%), threshold increases by >1 V (0.3–1.8%), and loss of left bundle branch capture (0.3–11.5%). The procedure can also be challenging in patients with septal scarring.

In our case, we identified a localized mid infero-septal scar, evidenced by late gadolinium enhancement on cardiac MRI. This finding is most likely due to a hinge effect of the lead interacting with the surrounding myocardium or an arteriolar injury during implantation. Interestingly, Hopman *et al.*^13^ recently reported a case of loss of capture caused by fibrosis surrounding the intraseptal lead, possibly resulting from a wear-and-tear effect. Note that experience with scarring on cardiac MRI following intraseptal lead implantation is limited, and the prevalence of fibrosis in these cases has yet to be documented, warranting further dedicated studies. Whether this fibrosis represents a benign physiological response or a precursor to complications, such as loss of capture or proarrhythmogenic events, remains unclear. Further research is needed to clarify the clinical significance of this imaging finding and its impact on patient outcomes.

We also observed frequent PVCs, originating from the same region as a limited antero-basal ischaemic scar. Treatment with flecainide and a beta-blocker was initiated, effectively suppressing the PVCs. Although flecainide is generally contraindicated in ischaemic heart disease,^14^ recent studies have demonstrated its safety in post-myocardial infarction patients with limited scar and no critical coronary artery disease.^15^ Importantly, neither the PVC burden nor the BFB was involved in the syncope of our patient. In this case, with poorly controlled Type II diabetes, further investigations revealed that severe orthostatic hypotension due to autonomic neuropathy was the underlying cause of the syncope. As emphasized, it is crucial to consider the differential diagnosis of syncope in the context of BFB, which raised a strong suspicion of arrhythmic syncope. Our patient had experienced recurrent syncope over the past 5 years, primarily triggered by positional changes. A thorough collection of clinical history was key in distinguishing between orthostatic syncope and arrhythmic syncope. By carefully examining factors such as the circumstances surrounding the syncopal episodes, the presence or absence of prodromal symptoms, and the patient’s medical history, we were able to identify autonomic dysfunction as a likely cause. Consequently, lifestyle modifications, dietary adjustments, and compression stockings were recommended. A clinical follow-up was performed 2 months after hospital discharge. Due to continued syncopal episodes while standing, fludrocortisone and midodrine therapy were proposed.

## Conclusion

Pacemaker implantation can effectively address syncopal episodes caused by paroxysmal AV block in the context of BFB. However, syncope may arise from multiple concurrent factors requiring a comprehensive evaluation. Left bundle branch area pacing effectively captures the left conducting system by implanting. However, this approach may result in local fibrosis due to trauma at the implantation site. Whether the surrounding scar tissue is proarrhythmic remains an area requiring further investigation.

## Data Availability

The data supporting the findings of this study are available from the corresponding author upon reasonable request. Due to privacy and ethical considerations, patient-specific data may not be publicly accessible but can be provided in anonymized form where applicable. Further guidance on accessing the data can be obtained from the corresponding author.

## Lead author biography

Betim Redzepi, a specialist in Internal Medicine and Cardiology, currently holds the position of chief resident in the Department of Cardiology at the University Hospital of Lausanne. Additionally, he is currently engaged in a fellowship programme specializing in cardiovascular imaging at the cardiovascular magnetic resonance centre of the University Hospital of Lausanne.

## Notes

**Consent:** Written consent has been obtained from the patient in line with the COPE guidance.

**Funding:** No funding was provided for this article.
